# A Qualitative Exploration of Aged-Care Residents’ Everyday Music Listening Practices and How These May Support Psychosocial Well-Being

**DOI:** 10.3389/fpsyg.2021.585557

**Published:** 2021-03-05

**Authors:** Amanda E. Krause, Jane W. Davidson

**Affiliations:** ^1^The Melbourne Conservatorium of Music, The University of Melbourne, Southbank, VIC, Australia; ^2^Department of Psychology, James Cook University, Townsville, QLD, Australia

**Keywords:** everyday music listening, well-being, older age, aged-care, agency, listening technologies

## Abstract

Strategies to support the psychosocial well-being of older adults living in aged-care are needed; and evidence points toward music listening as an effective, non-pharmacological tool with many benefits to quality of life and well-being. Yet, the everyday listening practices (and their associated specific psychosocial benefits) of older adults living in residential aged-care remain under-researched. The current study explored older adults’ experiences of music listening in their daily lives while living in residential aged-care and considered how music listening might support their well-being. Specifically, what might go into autonomous listening activities? 32 Australian residents (aged 73–98) living in two Australian care facilities participated in semi-structured interviews. The results of a qualitative thematic analysis revealed three themes pertaining to “previous music experiences and interest,” “current music listening,” and “barriers to listening.” While an interest in and access to music did not necessarily result in everyday listening practices, of those participants who did listen to music, perceived benefits included outcomes such as entertainment, enjoyment, relaxation, and mood regulation. Drawing on Ruud’s notion of music as a “cultural immunogen” supporting well-being and Self-Determination Theory, theoretical implications of the findings are addressed, relating to how to create and support music activities in aged-care facilities so that they are engaging, meaningful, and promote emotional regulation, community, and well-being.

## Introduction

Globally, average life expectancy is increasing (GBD 2017 DALYs and HALE Collaborators, 2018). As the population ages, health care demands drastically increase (Cubit and Meyer, 2011). Medical models have traditionally been the focus of health and disease management for older adults; however, approaches must also address the many social and emotional concerns associated with older age. While aged-care offers support for physical health through medical-model care, older adults living in these facilities are also offered social support through opportunities to continue participating in activities that they enjoy, to make life worth living and to aid well-being. The World Health Organization emphasizes “active aging” as a pertinent direction for health care policies, which refers to “the process of optimizing opportunities for health, participation and security in order to enhance quality of life for people as they age” (World Health Organization, 2002). Recent research suggests arts and cultural activities assist with active aging (Cann, 2017), as they help to promote quality of life and self-actualization. In particular, engaging in music has been identified as a promising activity for enhancing the quality of life of older adults, playing a vital role in supporting well-being for those both living in the community and in care facilities (Fraser et al., 2015; Cann, 2017).

### Music and Well-Being

Well-being encapsulates key components such as one’s satisfaction in life and the presence of positive emotions (Ryff and Keyes, 1995; Diener et al., 2009). When considering older adults and residential aged care, concerns regarding well-being include the transition into residential aged-care, which is often stressful and anxiety-provoking for older adults (Cheek et al., 2007; Ellis, 2010) and results in a loss of independence (Cheek et al., 2007) and control over their lives (Brownie et al., 2014). When living in aged-care, experiences of isolation, loneliness, and depression are also common due to declining physical condition and social changes associated a move from familiar surroundings (Aged and Community Services Australia, 2015; Australian Institute of Health and Welfare, 2017; Grau-Sánchez et al., 2017). With this in mind, it is important to consider how music can play a role in supporting the well-being of older adults. Ruud(2017, p. 589) theorized that music can act as a “cultural immunogen,” in that it “protects, promotes, and maintains our health and quality of life.” Ruud states that music engagement can contribute to four aspects of health: a sense of vitality, a sense of agency, a sense of belonging, and a sense of coherence and meaning (Hargreaves et al., 2017; Ruud, 2017). Indeed, numerous studies espouse the positive influence that music has on well-being in older age (Laukka, 2007; Fraser et al., 2015; Cann, 2017; Grau-Sánchez et al., 2017; Krause et al., 2018). To understand the impact of music engagement on older adults’ well-being, it is important to consider two key forms of music engagement: music-making and music listening (Vanstone et al., 2016; Krause et al., 2020).

#### Music-Making

Humans have been making music for millennia (Brown et al., 2000). This activity has ranged from self-generated vocalization and rhythmic patterning to highly elaborated collaborative outputs, and, as technologies have developed, instruments of various forms have requiring skilled plucking, striking and blowing actions in order to work. Coupled with the capacity to make musical sounds has been the ability to create musical outputs that might be spontaneous and improvisatory or else, highly planned, structured and notated. Amongst older adults, in addition to cognitive benefits and motor skill acquisition (Seinfeld et al., 2013; Schneider et al., 2019), music-making has been associated with increased well-being, as it can promote social interaction with others and provides a sense of purpose, autonomy and belonging, ultimately increasing life satisfaction and quality of life (Davidson, 2011; Creech et al., 2013; Vanstone et al., 2016; White, 2016; Grau-Sánchez et al., 2017). It has also been associated with a decreased reliance on medications and doctor’s visits to support health, as well as an increase in self-reported health amongst older adults (Cohen et al., 2006; Coffman, 2008; Hanna-Pladdy and MacKay, 2011; Hanna-Pladdy and Gajewski, 2012; Johnson et al., 2013; Noice et al., 2014; White, 2016). Creech et al.’s (2013) research similarly showed that participation in making music leads to higher levels of subjective social, emotional and cognitive well-being. This suggests that group music-making may indeed support a sense of purpose, control and autonomy, and social affirmation for older adults. Yet, for many older people, the opportunity for and access to music-making is limited (Davidson et al., 2014).

#### Music Listening

Older adults listen to music in order to reminisce, experience enjoyment, regulate moods, feel socially connected, and to express identity (Laukka, 2007; Hallam et al., 2012; North and Hird, 2020), and research suggests that older adults’ listening strategies correlate to perceived well-being (Laukka, 2007). Indeed, it has been shown that older people not only feel that the general importance of music increases in their lives post-retirement, but also that its meaning grows, especially in terms of self-regulation (Saarikallio, 2011, p. 317). Music listening evokes positive emotions (Västfjäll et al., 2012) and alleviates feelings of depression, anxiety, and stress (Västfjäll et al., 2012; Costa et al., 2018). Physical benefits include mitigating pain sensations, which in turn, reduced the need for medical interventions (Mitchell and MacDonald, 2006), as well as reducing blood pressure (Updike, 1990), nausea and vomiting (Madson and Silverman, 2010), and pain-related distress (Huang et al., 2010). In addition, listening to music promotes social bonding, fosters a sense of belonging to a social group, and helps to establish a social identity (Schäfer et al., 2013; Krause et al., 2020).

Studies investigating music listening and well-being have commonly used participants’ preferred music to generate personalized playlists and create listening programs (Knox and MacDonald, 2015; Griser et al., 2016). While Costa et al. (2018) work with older adults living in residential aged care demonstrated that a preferred music listening program can help relieve the symptoms of depression and anxiety as well as promote relaxation, positive reminiscence and emotional arousal, most listening research has focused on the benefits of listening for people with dementia (Davison et al., 2016; Griser et al., 2016; Garrido et al., 2018; Murphy et al., 2018; Kulibert et al., 2019; Weise et al., 2019). Research findings demonstrate benefits with regard to the symptoms of dementia as well as personal well-being. For example, Garrido et al. (2017) study demonstrated that reductions in agitation, anxiety, behavioral symptoms and depression were particularly evident compared to control groups. Also, older adults with dementia living in residential aged care gained cognitive benefits including improvements to memory (Elliott and Gardner, 2018), and improved interactions (Elliott and Gardner, 2018). Additionally, Pérez-Ros et al. (2019) demonstrated that participating in a preferred music-based intervention leads to improvements in the activities of daily living. Given a lack of such studies concerning older individuals without a diagnosis of dementia, it is important to investigate whether these effects can apply to these solo or group activities. Indeed, further research into the benefits of music listening for older adults without a diagnosis of dementia living in residential aged-care is necessary.

With listening, it is important to acknowledge the role of technology (Krause and North, 2016). Mobile devices, laptops, tablets, smart phones and streaming services are becoming increasingly popular for music listening, even amongst older adults (Krause et al., 2015; Edison Research, 2017). In spite of the common view that older adults find it challenging to use modern technology (Vines et al., 2015), they are capable and open to using technology to enhance their quality of life (Sixsmith and Gibson, 2007; Heinz et al., 2013; Creech, 2019). Older adults use (and can learn to use) technology in order to access their preferred music (Sixsmith and Gibson, 2007; Lancioni et al., 2014; Davison et al., 2016; Griser et al., 2016; Creech, 2019).

### Research Aim and Questions

The review of literature highlighted a significant gap in understanding the music listening experience for people living without dementia in residential aged care. It is important to understand this phenomena because older adults often listen to music (Laukka, 2007), and this common activity is embedded in everyday life (Schäfer et al., 2013; Krause et al., 2015), regardless of dementia diagnosis, music therapists, or formal interventions. Music listening can take place on its own, without some of the barriers associated with music-making and therapist-led interventions, such as the involvement of a facilitator, need for instruments, and training (Davidson et al., 2014). Thus, it is important to understand what is most suitable and what could offer the best possible well-being outcomes with regard to music listening in aged care. However, we first need to know what people are doing in this particular environment. Therefore, the current, exploratory study aimed to investigate older adults’ everyday listening practices and to explore how everyday music listening might influence quality of life for older adults living in residential aged-care facilities. In particular, the research addressed the following questions:

RQ1: How does music listening fit into the daily lives of older adults living in residential aged-care?
RQ2: How do older adults access music? What is the role of technology (e.g., radio, tablets, etc.)?
RQ3: How does music listening influence older adults’ perceived psychosocial well-being?

## Materials and Methods

### Sample

Thirty-two individuals living in two residential, aged-care facilities in the regional Melbourne area (75% female, 25% male) participated in one-to-one interviews. This gender representation reflected the overall population of the facilities. Of those who provided their age (90.63% of the sample), participants were between 73 and 98 years of age (*M* = 86.86, *Mdn* = 87.00, SD = 5.75), and most of the sample was Australian (or Australian and British, 87.50%), with the remaining individuals reporting their nationality as Canadian, English, Polish, and Dutch (one participant did not report).

Participation in the study was voluntary and open to residents. Any individual residing in the facility who could participate in an interview conducted in English was considered a potential interviewee, with no explicit research inclusion/exclusion criteria regarding length of stay, level of involvement in facility activities, or individual care needs. As part of the ethics protocol for the study, aged-care facility staff involved in the facility’s lifestyle activities made initial approaches to residents to explain the study and ensured that participation requirements were understood. Researchers then followed up with invitations to individual residents. After consultation with residents and staff about how to remunerate the participants for their time, researchers made a donation (valued at $25 AUD per participant) to support the lifestyle programming.

### Design and Procedure

The human ethics committee at The University of Melbourne granted approval for this study (Ethics ID: 1851094.2). A qualitative approach was used to examine the role of music listening in everyday life and how music listening might influence perceived quality of life. Semi-structured interviews were chosen as a data collection method because they are a practical/useful qualitative approach when researching older adults (Klein and Parks, 2007; Tkatch et al., 2017) and because it aligns with calls for selecting such research methods that facilitate our understanding of aging and everyday experiences (e.g., Kelly, 2010; Phoenix, 2018). Key interview questions were derived prior to conducting the interviews, which were conducted using a conversational style (such that these interviews can also be classified as informal, open-ended interviews; Bhattacharya, 2017). This meant that, in practice, each interview involved researchers and participants engaging in an informal, casual conversation exploring each participant’s personal experience (Bhattacharya, 2017).

The focal questions included in the interview schedule explored (1) people’s current music listening routines (specific preferences, how music is accessed, when and why people listen) and (2) their previous experiences with music-making and music listening throughout their life. Additionally, questions centered on people’s (3) weekly routines and how music might fit in, as well as (4) their lived experiences of the residential aged-care facility. As such, the data obtained was based on participants’ reflections of their personal experiences.

Participants were provided with information about the study, and were informed that they could discuss their participation with family, friends, and staff if they wished before consenting to take part. Individuals provided written consent prior to the interviews taking place. Individuals stated their age, gender, and country of residence on the consent form in order to report on the demographic details of the sample (no measures of physical or cognitive health were taken). All interviews took place in the aged-care facility at a time agreed upon by the individual participants.

### Data Analysis

With the consent of the participants, all interviews were audio recorded. Each recording was transcribed verbatim. Following Braun and Clarke’s (2006) six-step procedure, a thematic analysis was used to analyze the interview data. Braun and Clarke’s (2006) six steps involve initially familiarizing oneself with the data, coding data, collating recurrent, potential themes, checking the themes throughout the dataset, creating theme labels and definitions, and, lastly, reporting the findings using the themes and representative quotes. In particular, a reflexive, recursive approach was used: this permitted a flexible examination of the participants’ contributions without a focus on any particular theoretical background. Initial codes were generated first by identifying semantically similar responses from the entire dataset; then these codes were clustered in order to create broader, tentative themes. Although the formulation of themes was guided by semantic similarities, the coding of implicit concepts was also explored and included where relevant. Finally, higher order themes and sub-themes were refined and finalized in order to best represent the data with respect to the research questions. Names and pseudonyms have not been used in the reporting of the results in favor of increased participant confidentiality; however, quotes are tagged with a numeric participant code (participant = P).

## Results

Three overarching themes arose from the thematic analysis. These themes were labeled “previous music experiences and interest,” “current music listening,” and “barriers to listening.” These overarching themes and their sub-themes are summarized in Table 1 and are further considered below, supported by participant quotes.

**TABLE 1:** Summary of the emergent overarching and sub-themes.

Overarching theme (level 1)	Level 2 sub-theme	Level 3 sub-theme	Brief theme description
Previous music experiences and interest	Music-making		Earlier interests and experiences involving music-making (e.g., singing, playing piano, etc.)
	Music listening		Earlier experiences and interests in music listening (e.g., listening to the radio, having a music collection)
	Social dancing		Attending social dances (e.g., courting and meeting partners through dance involvement)
	Influence of the past on the present		Distinguishing current music experiences and interest from the past – evidence of interest waning, continuing, or waxing
Current music listening	Practices		Everyday listening behaviors (e.g., how and when people listen to music) – acknowledging that not all music encountered is self-initiated
	Preferences		Individual listening preferences (e.g., preferred artists, genres, instruments, radio stations)
	Perceived outcomes		Outcomes and benefits of listening to music (e.g., entertainment, enjoyment, relaxation, mood regulation)
Barriers to listening	Access and opportunities	Absence of (compatible) listening technologies	Acknowledgment that compatible devices are needed in order to play/listen to music (e.g., a radio that works in the facility)
		Access = / = use	The presence of music listening technology is not enough to result in listening (e.g., sometimes even though listening devices were present, people did not use them)
		Self-efficacy	People’s beliefs about their ability to use listening devices
		Physical limitations	Physical limitations (e.g., lack of mobility, dexterity)
		Watching TV	Watching TV is engaging, accessible
	Facility noise		
	Facility activities	Resident awareness	Individual awareness of facility-provided music-related activities (e.g., knowing what’s on)
		Personal preference	Individual preference for the types of facility-provided music-related activities

### Previous Music Experiences and Interest

The first theme concerned the participants’ experiences and interest in music prior to living in residential aged-care. During the interviews, residents spoke about their childhood, adolescence, and adulthood (though some participants focused on certain time periods, such that not all participants provided a full life history). This theme is divided into four sub-themes: music-making, music listening, social dancing, and the fact that people’s past experiences do not necessarily relate to their present music experiences or interest.

#### Music-Making

To begin the interview and introduce the topic of music experiences and interest in their lives, participants were asked about previous musical experience using a purposely broad question to embrace everything they might consider from formal music learning to informal or occasional social engagement involving music (see Krause et al., 2019a, for the wide-ranging conceptions of musical engagement). People responded in a range of ways, commenting on singing in a school choir, hearing their mother play piano at home, attending social dances, collecting records and attending concerts, supporting grandchildren’s music lessons, and attending musical activities at the facility, for instance. Half of the participants mentioned they had some sort of previous experience with music-making themselves, most commonly described as singing in the school or church choir (*n* = 12) or learning to play the piano (*n* = 5). Most of this previous experience was limited (P10: “*Oh, I was in a choir at school*”; P22: “*at school we used to learn music, but I didn’t like it*”). Only three participants spoke about a longer-lasting involvement with music-making. One woman learned piano and violin, earning a music degree and assisting/accompanying music groups as an adult (P05), one woman’s husband was a piano tuner who was also in bands and so she recounted how “*there’s been music in my life all the time really*” (P09), and one man had been in choirs from childhood through later life (P03: “*I’ve been involved in choirs in Holland, and also when I came to Australia …… I was also at [the University of the Third Age which] had a choir in Frankston and it became a choir of 100 members*”). A few more participants expressed that they had a desire to learn an instrument when younger, but that their family circumstances did not facilitate doing so (e.g., that the family “*didn’t have the money for that*”). Some mentioned family members who were musically inclined – often mentioning a mother or grandmother who played the piano (e.g., P14’s mother “*played the piano beautifully by ear*”), though a few other instruments were mentioned (P16’s brother played the accordion; P09’s father played the trumpet in a Melbourne orchestra).

#### Music Listening

In contrast to the limited music-making experiences, engaging in music listening activities was far more common in the participants’ lives. Twenty of 32 participants stated that music listening activities featured in their life (at some stage) prior to residing in the aged-care facility. Participants mentioned listening to the radio, as children (P29: “*we listened to the radio and I can remember stories, … and kind of having my ear more or less glued …to the radio*”) and as adults (e.g., P25: “*I used to love music. We had a lovely radiogram …and I just loved it*”), and of having music collections. Collections involved records, cassette tapes, and CDs (e.g., P09: “*I had a lot of records*”; P06: “*too many records*”). A small number of individuals also mentioned going to concerts, musicals, and theater performances (P08: “*In my heyday I [went to] all the symphony concerts and I used to go to them in Melbourne Town Hall*”). Attending dances was another way participants mentioned experiencing music, with one participant saying that she could not get records because she was living with other people, but was able to hear music by going to dances.

#### Social Dancing

The presence, and role, of social dancing is an interesting sub-theme. Of the 18 discussions about social dancing, only four people stated that they did not go to dances (the subject was not raised in the other 14 interviews). ‘In other words, social dancing featured in many of the residents’ lived experiences (P27: “*I never missed a dance*”; P10: “*I loved the dances …that was my main interest, I think, was dancing*”). Even for those who stated that they were not very much interested in music, dancing was an important social activity. Participant 04 stated,

“I always went to dances from about 14 on, because we had a woman come and taught us dancing, because as I said, my father was an emcee. On Thursday night and Saturday night, we went to both dances whether we liked it or not. There was a woman there that could teach dancing …I loved dancing from then on. It was great when you were at sea because every time you went into port, you went to a dance.”

For some of participants, dancing and dating went hand in hand (e.g., P07: “*Oh, yes. There was the dancing. Yes, it was dancing. They invite me, the boys. Yes. I like dancing*”) – both Participant 09 and 17 met their husbands at a social dance.

#### Influence of the Past on the Present

Interestingly, participants’ past music experiences did not seem to relate to their current music experience or interest in music. People distinguished their past music experiences from their present “relationship” with music; and the evidence shows a variety of patterns for changes in these relationships throughout life. For instance, the two participants with arguably the most musical experience throughout their life presented contrasting levels of engagement and interest at the time of the interview. The life-long singer (P03) continued to seek out musical activities, and, while he recognized he was “*not as active as [he] used to be in music*,” he stated that he had “*started doing some songs with some of the older ladies in the 80s, 90s and … they just love it*” and was “*trying to organize [his] son to get [him] a record player*.” The woman with a music degree (P05), on the other hand, “*hasn’t kept playing*” but stated that she does attend the facility concerts – plus, while she had a collection of cassettes and CDs in her room, she said she watched the television more than listening to music. Other participants who previously sang or played the piano as a child finding enjoyment in it stated they did not listen much at the present time (e.g., only “*now and again*” – P17) or did not “get around to it” often. The reverse pattern was also present, Participant 21 did not learn an instrument or seek out much music when younger, but made use of a radio in his room, leaving “*it on all day*.”

### Current Music Listening

Three sub-themes arose with regard to the participant’s current music listening experiences: people’s practices, preferences, and the perceived outcomes.

#### Practices

A number of participants did listen to music in their everyday lives (44% of the sample), though these individuals spent different amounts of time listening. For instance, Participant 21 used a stereo (radio) in the room daily, listening to a radio station that plays light classical music (“*a bit of light, soft music, yeah. I just leave it on all day*”). Participant 32 listened “*most days*,” drawing on her cassette tape collection, while participant 01 estimated he listened to the radio 2–3 h per week. In contrast, Participant 17 listened “*now and again*.” Participants drew on a range of formats and devices to listen to music – the radio, stereo, cassettes, CDs, DVDs, the TV, and tablets were all used (and often on display in people’s rooms). Fifty-six percent of the sample had some sort of listening technology in their room. Additionally, some participants (38%) mentioned facility lifestyle activities (i.e., concerts and sing-alongs) as ways they listened to music while living in residential aged-care.

Other participants acknowledged they did hear some music in their daily lives, although it was not necessarily sought after. For instance, Participant 30 stated “*I love music, I like music around, but I don’t go out and put it on*” and Participant 02 stated “*I wouldn’t think of it doing that myself*.” While these listening opportunities were not instigated by the participants themselves, the music encountered was enjoyed. For example, Participant 14 said that “*all the music that comes over here from the dining room is very pleasant*,” expressing enjoyment from the music she experienced in her environment, without actively contributing herself. In other words, some participants listen to music “*when it’s played*” (P13) by others in the facility.

Of course, although music listening is a common leisure pursuit, not everyone is interested. This lack of interest was present in some of the participants in the present sample, as a few participants stated that they did not have an interest in listening to music in their daily life. For some this was in line with a longer-standing relationship with music (e.g., P28: “*never been a great radio listener*”). For others, it was not something they disliked, rather something that it simply was not prioritized (e.g., P08: “*No, I don’t seem to get around to it*”).

#### Preferences

Participants’ mainly conveyed their listening preferences via broad genres and particular artists. While there was individual variation in the type and range of music enjoyed, most participants favored classical and popular music. Classical composers referenced included Mozart, Beethoven, Vivaldi, Bach; mentioned performers included the Three Tenors and André Rieu (though Rieu was not universally enjoyed); and some clarified their classical music interest in terms of instruments (e.g., piano, violin) or radio station (e.g., the local light classical station). Most participants enjoyed popular music from earlier in their lives (“*all the old songs*,” P14). For many, this included musicals (e.g., *On the Town*) and operettas (e.g., works by Gilbert and Sullivan, like *Pirates of Penzance*) and “*all the popstars*” (P25), such as Frank Sinatra, Doris Day, Elvis and others from the movies and “*the great musical comedies like Annie Get Your Gun and My Fair Lady*” (P02).

Some participants also enjoyed country and western music, referencing Roy Orbison, Slim Dusty, Jim Reeves, as well as Australian Folk music (e.g., the Bushwhackers). Some also expressed enjoying dance band and jazz music (e.g., Glen Miller, Tommy Dorsey), which often was associated with previous social dance experiences. Lastly, *The Goons*, a radio comedy program also received one mention.

#### Perceived Outcomes

In some (but not all) interviews, natural discussion arose relating to the perceived outcomes or benefits of listening to music in everyday life. Participants referenced outcomes including entertainment, enjoyment, and relaxation, as well as benefits including mood regulation and aiding worship. The highly engaged life-long singer (P03) was aware of the well-being benefits of engaging with music: he declared that “*music is – it’s like a medicine*.” However, not every participant spoke with such clarity. For instance, after Participant 06 stated that she loves music, she was asked what it is she likes about it and replied “*I enjoy it*” without further explanation. Similarly, to the same sort of follow-up question, Participant 12 replied “*I can’t tell you. I just like it*.”

The participant who made use of her cassette tape collection to listen to music in her room (P32) explained that she has “*lay back time*” on most days, stating that the music relaxes her. Thus, in addition to enjoying the music listening or fighting boredom, this participant actively used music for relaxation. Music was also to assist some in falling asleep (P10).

As in previous research, participants also used music listening to regulate mood. Participant 23 stated she would “*cheer [her]self up with something*” and Participant 25 referenced times when she would turn to music to let go of frustrations. Additionally, with a radio in her room set to a local Greek station, Participant 22 was able to listen to music and virtually attend church services in her native language.

### Barriers to Listening

A number of barriers to regularly listening to music in residential aged-care were identified in the interview conversations. The barriers mentioned pertained to access and opportunities (further divided into five sub-themes), facility noise, and facility activities (further divided into two sub-themes).

#### Access and Opportunities

The primary barrier to listening to music in aged-care concerned access and opportunities. This is evident from the data with regard to the fact that not everyone had technologies that would facilitate music listening and that, for those who did, access to technologies and recorded music collections did not align to their usage. Related issues concerned self-efficacy with listening technologies, physical limitations, and competition for engagement by the TV.

For some residents, one barrier was the absence of (compatible) listening technologies. When asked, Participant 20 stated that her “*room doesn’t have the ability to play music*.” While debatable, such a seemingly simple declaration has nuance, when interpreted relative to other facility residents’ experiences. It illuminates differences in opportunities that can arise depending on residents outfit their individual spaces. While it is, of course, possible to play music in residents’ rooms, that action depends on having the technology. For some residents, securing such technology is not easy. For instance, participant 04 stated, “*I’ve often thought I should buy myself a radio because of music, but I haven’t got around to doing it because I can’t get out. So …someone’s got to buy it for me*.” While family could assist in the acquisition of listening technology (P03: “*I’m trying to organize my son to get me a record player*”), family members can also take it away: Participant 24 did not listen to the radio because her daughter took it in an effort to decrease “clutter” (“*my daughter just yesterday, believe it or not, she just took the radio home. She’s having a clean out*”).

Further, any technology must also be facility-compatible. Participant 25 recounted that, “when I came in, John went out and bought me a digital radio and it wouldn’t work and he got the maintenance man to come and have a look. He said, “is that digital?” Jack said “yes,” he said, “it won’t work here.” Jack said, “why ever not”? He said because of all the electronics. He said “there’s no room for digital.” He said “you’ll have to do something with it and … go and get another one because this one won’t work.””

However, the data does not suggest that the presence of opportunity is enough. Just as previous experience or interest in music did not necessarily result in present listening, the presence of listening technologies and recorded music collections did not necessarily result in the usage of said technology or collections. Many of the participants had listening devices (such as radios, stereos, and tablets) and collections (such as cassettes, CDs, and records) in their rooms. Yet, even the collections visibly on display were not indicative of regularly daily listening. Participant 02 remarked, “*we’ve got all those CDs and there’s records down below. There’s tapes over here which we used to listen to much more before we came here*.” She goes on to acknowledge she and her husband sometimes thought about engaging with their listening collection (“*we sometimes [would say], we should listen to those more*”), but that their lifestyle had changed, shifting leisure pursuits.

For some participants, use of their listening devices depended on their level self-efficacy (an individual’s belief in their capacity or confidence in doing something). In particular, using tablets (often provided by family members) presented challenges. For instance, Participant 30’s daughter set up a tablet with Spotify for her, but she stated that she did not use it, saying “*I’m not very good at these sort of things*.” Participant 23 also had a tablet, and similarly stated she had “*to keep getting somebody in to help*.” References to a lack of confidence more often concerned the use of digital devices, though self-efficacy concerns were raised with other media as well. For instance, Participant 03 listened to the news on his radio, but did not use the radio for music because he did not know how (“*I don’t know how to do it, so I haven’t found out yet*”).

Other participants faced physical limitations with regard to engaging in music. A lack of physical mobility and dexterity prevented individuals from listening, even when the desire was present. For instance, Participant 09 had a collection of tapes, CDs, and DVDs in her room but did not use them: “*I never – I haven’t watched any of this since I’ve been in here, because I can’t get to the machine. That’s the trouble. Same with the tapes I’ve got. …I really love music. It’s just a pity that I can’t get to them*.”

It is also worth noting that such physical limitations also present barriers to participating in musical activities supported by the care facility.

P03: “*When there’s a choir coming here, I attend, and if I know the songs, I try to sing with them. But because my eyes are no good, I can’t read the words, it makes it difficult*”
P05 had an accident, altering her ability to engage with music: “*I’m lucky I can talk. But I can’t sing anymore. But I take …a tambourine and I do percussion now [at the sing-along sessions]*.”
P08 desired to play the piano in the facility lounge, and found it hard because of her memory problems: “*I moved to Melbourne and came into this place, and of course they’ve had times when they needed a pianist but they haven’t had one. But it’s terribly hard to hear a need and then not be able to*.”

Such examples reveal how, for some people, physical limitations will overshadow an interest in music. In other words, declining mental and physical health pose real challenges for older adults living in residential aged-care to engage in music activities.

A final barrier concerns the consideration of listening to music relative to watching TV. Even for many of the participants who had listening technologies in their rooms, the TV appeared to be more engaging (P05: “*I’ve got a radio there, my son fixed it up. I do, but I tend to watch the television more*”). Most of the participants expressed a greater use of the TV in daily life (P26: “*I watch television more than I suppose anything else. Particularly being here”*; P25: “*everything’s on the telly anyway*”). Interestingly, only one participant (P16) mentioned that they could access music on the TV – for the others, this did not seem to cross their mind. For instance, Participant 04 had Foxtel (a cable provider), but when asked about using the cable TV to listen to music via the dedicated channels, he had not considered it (“*I haven’t even looked, no*”). The TV may offer untapped opportunities for music listening as the use of it as a tool is highly familiar to all participants, even though its technological potential is not fully understood or explored.

#### Facility Noise

Living in aged-care facilities presented a barrier to music listening with regard to noise. This is best exemplified by Participant 23 who said she did like to listen to music, but “*not here, though. I can’t stand it. Because the noise here is driving me mad. … I’d only hear bits and I think it’s annoying me. So I tried it but there were so many interruptions*.” Other participants also did not want added noise. Participant 22 also stated she would not be interested in listening to music “*because I don’t want too many noises*.”

#### Facility Activities

Some participants acknowledged that the facilities provided some opportunities for music listening – often in the form of concerts and sing-alongs.

P04: “*We have music every week here. We have a lady [who] comes in and plays the piano and she’s wonderful. I go out and sit and listen to her*”
P05: “*I go to all the music things*”

Yet, two barriers concerning the facility offerings were identified: resident awareness and preference. Notably, the participants’ level of awareness of music-related activities greatly varied. When asked if she attended concerts, Participant 17 said, “*They don’t have concerts here. I don’t hear of them anyway. … I go if I know it’s on*.” Her response reveals that this barrier is not due to a lack of interest, but rather the level of involvement and/or cognitive functioning of individual residents. The other barrier concerns the potential difference between personal preferences and what is offered. For instance, facility staff played music in a common, lounge space, but the music selected may not suit every individual. Participant 11 provided a nice example: “*what is often played here is André Rieu. Everybody else loves him but we don’t*,” suggesting that it is important to offer a variety of music (and activities).

## Discussion

The present study explored older adults’ experiences of music listening in their daily lives while living in residential aged-care. Results of a thematic analysis of the semi-structured interview data highlight a difference between people’s previous musical experiences and interest with present music listening practices and a number of barriers to everyday listening. While the majority of participants may not have actively created daily music listening opportunities for themselves, they largely enjoyed the listening experiences they did encounter. Although previous work on listening technologies has indicated that listener choice and control is associated with positive outcomes (e.g., Krause et al., 2014, 2015), it appears that, for these participants who were older adults living in residential aged care, positive outcomes can also result from music heard that is not personally sought after (noting that personal preferences will also play a role in how the music is perceived – e.g., the participant who pointed out that what was played in the lounge did not suit their taste). This is encouraging when considering that, additionally, the present findings indicate that those who did listen to music spoke of the positive outcomes, such as benefits to their well-being.

Thus, the resulting themes and sub-themes concerning older adults’ everyday listening practices can be considered relative to Ruud’s (2017) notions of how music can promote four areas of health (namely, a sense of: vitality, agency, belonging, and coherence/meaning). People’s previous music experiences and interests, and the noted experiences with courtship via social dancing in particular, demonstrated clear examples of experiencing meaning, vitality, and belonging. Thus, it may be possible to draw on people’s previous experiences with music to promote meaning through nostalgia, given music has been shown to provoke nostalgia and autobiographical memories (Michels-Ratliff and Ennis, 2016; Garrido and Davidson, 2019). However, it is important to consider people’s experiences of meaning, vitality, and belonging from their *current* engagement with music. The perceived outcomes, or benefits, of listening to music that the participants mentioned indicate that, for some older adults, engaging with music is linked to these senses of vitality, belonging, and meaning. For instance, the highly engaged life-long singer’s (P03) identity was tied to his musical engagement, having participated in choirs throughout his life. While he stated he was “*not as active as [he] used to be in music*” at the time of the interview, he attended all of the facility’s sing-alongs and performances, suggesting he continues to support his identity and derive coherence and meaning via his continued musical engagement. Additionally, one interpretation of using music listening to promote relaxation, mood regulation, and sleep is that this particular *use* of music promotes vitality through promoting physical and emotional well-being. Further, there was evidence that listening to music was associated with the sense of belonging; for instance, one participant made use of a local community language radio station in order to continue participating in her Greek community.

With regard to agency, however, as the results indicate, previous musical experiences and engagement (both making and listening) do not necessarily translate into current, active listening practices. It appears that access is not enough to spur usage; rather, listening to music can be perceived as an effortful activity. Indeed, the identified barriers overshadow many older adults’ interest in and desire to include more music listening in their daily lives. In the context of Ruud (2017) view, then, *access* does not equal *agency*. As demonstrated, some individuals may need support – for instance to overcome physical limitations, bolster self-esteem, or a reminder of what is possible. As most of the sample enjoyed music listening and experienced known well-being benefits, the consideration of how to support listening for older adults living in aged-care is a worthwhile endeavor.

Importantly, well-being and quality of life are not fixed. As Ruud(2017, p. 593) stated, these concepts are in flux and can be influenced – thus, we can consider how music can offer a way to “mobilize oneself toward a better quality of life.” Research confirms that the benefits of engaging in music are wide-ranging (e.g., Krause et al., 2018), so how can the frequency of listening to music in residential aged-care be increased? How can aged-care staff and loved ones encourage their older adult residents to listen to music in order to support their well-being?

To answer such questions, the present findings can be considered with regard to meeting people’s basic psychological needs. As outlined by Self-Determination Theory^1^, meeting the three innate needs of competence, relatedness, and autonomy leads to personal growth, vitality, and well-being (Ryan and Deci, 2002; Milyavskaya and Koestner, 2011; Lombas and Esteban, 2018). Competence concerns being effective in one’s efforts; relatedness concerns feeling socially connected; and autonomy concerns feeling that your actions are self-governed and self-endorsed (Deci and Ryan, 2000). People’s basic psychological needs are fostered in autonomy-supportive environments (and hindered in controlling environments; Bonneville-Roussy et al., 2013): and, in aged-care, autonomy-supportive environments are associated with better well-being (Ferrand et al., 2014). With this in mind, it is important to consider how everyday listening practices might be used to assist people’s feelings of competence, relatedness, and autonomy (and what support might be needed to assist older adults’ in listening to music to promote their basic psychological needs).

The present findings most clearly link people’s access to, and self-efficacy concerning the use of, (compatible) listening technologies to the needs for autonomy and competence. Indeed, the noted barriers in the present study suggest that some older adults may need personalized support to bolster their everyday engagement with music. Technological support to available listening technologies could assist older adults’ with self-efficacy, and, thus, also expand their competence. Such support could be an assistant who works with the residents in learning to use listening technologies and/or modifications to the technologies employed. Handheld digital devices (i.e., tablets, mp3 players) can offer personalized selections and overcome the physical challenges associated with using physical media; however, the participants’ comments regarding self-efficacy suggest a switch to newer technology may not suit everyone. Facility-compliant radios were popular and easy to use. Additionally, the TV (e.g., via dedicated channels, satellite programming) should be considered as an alternative listening device that could increase daily listening. By offering the potential for both aural and visual stimulation, the use of TV raises interesting questions: it is prudent for future work to consider the potential importance of the visual component when designing engaging music listening opportunities in residential aged-care. TVs and other devices with touch screens (e.g., tablets) may offer older adults opportunities for engaging in audiovisual activities, while at the same time promoting technology skills.

Aged-care staff may also consider the variety of (recorded and live) music on offer (e.g., the range of music played in communal spaces). Providing residents with agency in choosing the music through consultation will promote autonomy. Such consultation could mean that residents’ who lack in competence could still feel a sense of agency and autonomy. Staff could make use of people’s varied past experiences and interests as a catalyst for planning purposes (e.g., promoting positive reminiscing of past dancing experiences). Moreover, drawing on residents’ personal histories and interests as well as including them in decisions will promote feelings of relatedness. It is important to note, however, that listening to self-selected music still has the potential for adverse reactions: for instance, given music can promote reminiscence, it can trigger distressing memories (Garrido et al., 2020). Thus, it is important to carefully prepare, create, and monitor music listening opportunities.

One strength of music listening is that it is a versatile activity: it can be private or shared and can happen at most times in most places. Indeed, even if someone is listening alone, music can promote feelings of belonging and community (e.g., Schäfer and Eerola, 2020). However, when considering how to promote feelings of relatedness, it is also worth considering how incorporating social interaction into music listening opportunities could be useful – in terms of both continued engagement and well-being. While social interaction is the central feature of sing-alongs and concerts formally offered as facility activities, residents must be aware of, and interested in, attending these. Music listening could be informally added to additional activities – for instance as background music to other leisure activities and meals and shared, in-room conversations. This could increase the prevalence of encountering music for those who may not feel they have the personal agency to do so (though the integration of background music would need to take into account issues such as hearing abilities and personal preferences). Additionally, educating staff, family members, and the residents about the potential benefits of music listening to well-being could further facilitate efforts to encourage older adults to listen. Knowledge of the benefits of listening might support transitioning an interest in, and access to, music into everyday listening practices. Such listening could be tailored to individual interests, and provide stimuli for conversation and reminiscence amongst residents, staff, and family members.

### Limitations and Future Directions

The present research is not without its limitations. Firstly, it draws on qualitative data from the focal questions from interviews with 32 individuals. Although the theme concerning the barriers to listening arose without direct prompt, the other themes speak to key questions such that other experiences may not have been explored in depth (e.g., the present focus on music listening rather than music-making). Moreover, the participants resided in just two aged-care facilities. Aged-care facilities differ in terms of their chosen care model and lifestyle activities on offer. Individuals’ music experiences and interests will vary, as will their physical and mental health care needs and existing social support structures. Drawing on the present findings, future research might overtly examine such related variables, drawing on quantitative measures as well as methodologies that utilize real-time data collection (e.g., experience sampling) to further our understanding of everyday listening experiences in residential aged care. Future research aimed at developing strategies to promote and facilitate music listening should take into account physical and mental health as well as the existing social support structures. Additionally, participants had resided in the facilities for varying lengths of time, and it is possible that this influences people’s daily practices, care experiences, and motivations. For instance, research suggests that transitions are often difficult for residents (e.g., Street and Burge, 2012; Lee et al., 2013), so future research could examine how to support the transition with music listening (given it’s frequently used to cope with stress – de Witte et al., 2020).

While the findings demonstrate that a range of technology is currently used to listen to music, more and more people entering residential aged-care in the future will have higher fluency and self-efficacy with using digital technology. This will likely shift the use, and role, of personal technology devices, including those used to listen to music. Given the rise in people using digital devices and listening to personalized playlists (Savage, 2016; Krause and Brown, 2019), more and more future residents may bring individualized listening habits with them into residential care. While concerns around noise in the facilities were raised, headphone use was not addressed in the present study; thus, future work is also needed to consider the use of speakers and headphones, keeping in mind issues around physical comfort and problems encountered owing to hearing loss for older persons. Further, while there is growing evidence of positive outcomes from music playlists in aged-care (e.g., Garrido et al., 2017; Thomas et al., 2017), future research still needs to consider alternative access and delivery options for music listening opportunities. As previously mentioned, it would be beneficial to consider how to make use of TVs, given the propensity of residents using TVs in their rooms on a daily basis. Additionally, because access did not necessarily translate into use, it is important to for future work to examine how best to create enabling and sustainable listening opportunities.

In summary, the present study examined the nature of everyday listening practices of older Australians living in residential aged-care facilities. Although the findings identified some barriers aged-care residents face to listening to music, residents largely enjoy listening to music and listening experiences offered opportunities for entertainment, relaxation, and interaction with others. When considering that music listening can support well-being, the findings have broad implications regarding how aged-care facilities might encourage and support residents to meaningfully engage with music in order to promote well-being.

## Data Availability Statement

The datasets presented in this article are not readily available because of the ethics conditions. Requests to access the datasets should be directed to AK, amanda.krause1@jcu.edu.au.

## Ethics Statement

The study involving human participants was reviewed and approved by The University of Melbourne – Human Ethics Committee (Ethics ID: 1851094.2). The participants provided their written informed consent to participate in this study.

## Author Contributions

AK obtained funding and ethics approval for the research, conducted the data analysis, which was then reviewed with JD, and wrote the initial version of the manuscript with JD offering later input. Both authors carried out the facility visits and interviews. Both authors contributed to the article and approved the submitted version.

## Conflict of Interest

The authors declare that the research was conducted in the absence of any commercial or financial relationships that could be construed as a potential conflict of interest.

## Footnotes

1 The Self-Determination Theory framework has been used previously to understand the relationship between musical engagement and well-being (e.g., Küpers et al., 2014; Evans, 2015; Krause et al., 2019b).
